# Episiotomy Use in a Low‐Income Setting: Prevalence, Associated Factors, and Maternal Outcomes Among Vaginal Births at a Tertiary Hospital in Ethiopia

**DOI:** 10.1155/bmri/9673092

**Published:** 2026-10-12

**Authors:** Nebyu Yonas Shanka, Biniyam Esayas Dana, Kidist Abrham Bosha, Daniel Desalegn Kuma, Bekalo Bekele Bale, Tigist Ewunetu Limeneh

**Affiliations:** ^1^ Wolaita Sodo University Comprehensive Specialized Hospital Wolaita Sodo Ethiopia

**Keywords:** associated factors, episiotomy, Ethiopia, maternal outcomes, prevalence, vaginal birth

## Abstract

**Objective:**

To estimate episiotomy prevalence, identify independently associated factors, and describe maternal outcomes among vaginal births at a tertiary hospital in Ethiopia.

**Methods:**

We conducted a retrospective cross‐sectional review of 306 sampled records of women with eligible vaginal births at Wolaita Sodo University Comprehensive Specialized Hospital from January 1 to February 28, 2023. Variables with a bivariable *p* < 0.25 were entered into a multivariable logistic regression model. Adjusted odds ratios (AORs) with 95% confidence intervals (CIs) were reported. Perineal outcomes were compared by episiotomy status using unadjusted analyses.

**Results:**

Episiotomy was documented in 141 births (46.1%). Higher odds of episiotomy were associated with no documented antenatal care (AOR 1.98, 95% CI 1.10–3.57), second‐stage duration of at least 1 h (AOR 2.70, 95% CI 1.52–4.81), postterm birth (AOR 9.73, 95% CI 1.92–49.16), and birth weight of at least 2500 g (AOR 3.46, 95% CI 1.25–9.57). Lower odds were associated with multiparity, documented comorbidity, preterm birth, and absence of meconium‐stained amniotic fluid. Documented second‐degree tears were more frequent among women with episiotomy than among those without it (22.7% versus 11.5%; unadjusted OR 2.26, 95% CI 1.21–4.19).

**Conclusion:**

Episiotomy was common and associated with several measured maternal and intrapartum characteristics. Because the maternal‐outcome comparison was unadjusted and susceptible to confounding by indication, it does not establish that episiotomy caused perineal injury. The findings support restrictive, protocol‐based use and regular institutional audit.

**Operational Definitions**

• Antenatal care: a package of preventive, promotive, and supportive health services provided by skilled health personnel to pregnant women to promote maternal and fetal well‐being and to support a healthy pregnancy and childbirth.
• Apgar score: a method used to assess a newborn′s condition immediately after birth based on five components: heart rate, respiratory effort, reflex irritability, muscle tone, and color. Each component is scored from 0 to 2, giving a maximum total score of 10. The score is usually assessed at 1 and 5 min after birth and provides an objective measure of the newborn′s adaptation to extrauterine life.
• Birth weight: the weight of a newborn measured immediately after delivery or as soon thereafter as feasible, expressed in grams.
  1. Low birth weight: a newborn birth weight of less than 2500 g.
  2. Very low birth weight: a newborn birth weight of less than 1500 g.
  3. Extremely low birth weight: a newborn birth weight of less than 1000 g.
• Episiotomy: also known as perineotomy, a surgical incision made in the perineum and posterior vaginal wall during the second stage of labor to enlarge the vaginal opening and facilitate childbirth.
• Instrumental vaginal delivery: a vaginal delivery assisted by the use of instruments, such as forceps or vacuum extraction, to facilitate the birth of the baby.
• Parity: the number of times a woman has given birth at or beyond 24 completed weeks of gestation, regardless of whether the infant was born alive or stillborn. This includes vaginal and cesarean births.
• Spontaneous vaginal delivery: a vaginal birth that occurs without the use of instruments such as forceps or vacuum extraction.

## 1. Introduction

Episiotomy is a surgical incision made in the perineum during the second stage of labor to enlarge the vaginal outlet and facilitate vaginal birth [1]. It has historically been one of the most frequently performed obstetric procedures, particularly among primiparous women and in operative vaginal delivery, based on the assumption that a planned incision could prevent uncontrolled perineal trauma, shorten the second stage of labor, and improve maternal and neonatal outcomes [2, 3]. However, these presumed benefits have been increasingly questioned, and the role of episiotomy in contemporary obstetric practice has been substantially reevaluated [2, 4, 5].

Current evidence favors restrictive rather than routine use of episiotomy. Studies have shown that liberal episiotomy does not consistently prevent severe perineal injury or improve neonatal outcomes and may instead increase maternal morbidity, including postpartum pain, blood loss, deeper perineal trauma, and anal sphincter injury [2, 4–6]. Current WHO maternal health recommendations state that routine or liberal use of episiotomy is not recommended for women undergoing spontaneous vaginal birth, supporting a restrictive and selective approach to its use [7]. Accordingly, major professional and international bodies recommend that episiotomy should be reserved for clear clinical indications, such as selected operative vaginal births or situations requiring urgent delivery, rather than used as a routine component of vaginal birth care [4, 8]. This shift from routine to selective practice reflects broader efforts to promote evidence‐based, respectful, and safer intrapartum care [4, 7, 9].

Beyond its immediate procedural role, episiotomy is important because of its potential implications for maternal outcomes. Previous studies have linked episiotomy to postpartum pain, delayed recovery, dyspareunia, wound complications, and extension into more severe perineal injury [2, 5, 6]. Evidence also suggests that episiotomy in one delivery may increase the risk of obstetric laceration in a subsequent vaginal birth, highlighting that its consequences may extend beyond the index delivery itself [10]. Because the procedure directly affects maternal well‐being and quality of postnatal recovery, evaluating its frequency and determinants remains an important component of improving the quality of childbirth care [6, 10].

The focus of the global discussion has also shifted. Early efforts concentrated on lowering episiotomy rates, but attention has since moved to what happens to the woman afterwards: how quickly the perineal wound heals, how much pain she has, how soon she resumes normal activity, and how her pelvic floor functions in the months and years after birth [5, 9, 10]. Contemporary work therefore looks beyond whether an episiotomy was performed and asks how the perineum is repaired, cared for, and followed up, because the wound environment itself influences healing time, infection, and scar quality [11, 12]. Long‐term concerns such as dyspareunia, urinary and anal continence, and pelvic organ prolapse have become part of how the procedure is judged [9, 10]. The quality of episiotomy care is therefore now measured by two things together: appropriate use of the procedure and the quality of maternal recovery that follows it [13, 14].

Despite increasingly restrictive recommendations, episiotomy practice continues to vary widely across countries and institutions [13, 14]. Marked international differences have been reported, reflecting variation in obstetric training, institutional protocols, case mix, provider preference, and the degree to which evidence‐based guidance has been implemented [15, 16]. Studies from Asia and Latin America have documented persistent overuse in some hospital settings despite policy shifts toward selective practice, whereas studies from high‐income settings have shown substantial reductions after guideline‐aligned quality improvement efforts [17, 18]. Within Ethiopia, available studies also indicate marked interfacility variation. Earlier hospital‐based evidence from Addis Ababa documented a considerable magnitude of episiotomy practice [19], whereas subsequent Ethiopian studies reported high prevalence and identified factors such as parity, duration of the second stage of labor, birth weight, and service‐related characteristics as important correlates of episiotomy use [20–22]. More recently, a systematic review and meta‐analysis from Ethiopia confirmed substantial disparity in episiotomy practice, particularly between primiparous and multiparous women, and underscored persistent national‐level variation [23].

The recently published study from Batu General Hospital provides an especially relevant Ethiopian comparator for the present manuscript [24]. In that cross‐sectional review of 300 vaginal deliveries, episiotomy was performed in 26% of births, and the procedure was strongly associated with vacuum‐assisted delivery and low first‐minute Apgar score [24]. This finding suggests that, within Ethiopian hospital settings, episiotomy may be more closely linked to intrapartum complications and higher risk deliveries in some facilities, whereas in others, it may remain more strongly influenced by routine practice patterns. The variation between Batu and other Ethiopian reports indicates that prevalence and determinants are likely to differ according to referral profile, case severity, provider practice, and institutional standards. Such variability limits direct generalization across facilities and underscores the importance of generating local evidence from additional comprehensive specialized and tertiary hospitals.

Wolaita Sodo University Comprehensive Specialized Hospital serves a large catchment population in southern Ethiopia, yet updated evidence on episiotomy prevalence, associated factors, and maternal outcomes in this setting remains limited. Because comprehensive specialized hospitals commonly manage referred cases, prolonged labor, instrumental births, and other obstetric complications that may influence perineal intervention, the determinants and consequences of episiotomy in such settings may not mirror those reported from lower level facilities or different geographic areas [22–24]. Generating context‐specific evidence is therefore important not only for quantifying the burden of episiotomy but also for evaluating whether its use reflects appropriate clinical indication or potentially modifiable overuse.

Accordingly, this study is aimed at assessing the prevalence of episiotomy, identifying associated factors, and examining maternal outcomes among women who gave birth at Wolaita Sodo University Comprehensive Specialized Hospital, Ethiopia. By providing hospital‐based evidence from a tertiary care setting, the study seeks to support efforts to optimize episiotomy practice, strengthen appropriate clinical decision‐making, and improve the quality and safety of maternal care during vaginal delivery.

## 2. Methods

An institution‐based retrospective cross‐sectional study was conducted at Wolaita Sodo University Comprehensive Specialized Hospital, Ethiopia, to assess the prevalence of episiotomy practice and factors associated with its use among women who delivered vaginally. The study included women who gave birth vaginally at Wolaita Sodo University Comprehensive Specialized Hospital between January 1 and February 28, 2023, and whose medical records contained complete information for the study variables.

The source population comprised all women who gave birth vaginally at the hospital during the study period. Charts of women who had instrumental vaginal delivery (vacuum‐assisted, forceps‐assisted, or destructive delivery), breech vaginal delivery, multiple gestations, or incomplete documentation were excluded from the study. The maternal outcomes assessed were perineal tear and its degree following vaginal birth, together with any other maternal complication recorded in the chart. The documented indication for each episiotomy was also extracted. Among women undergoing episiotomy, a perineal tear was recorded only when an additional spontaneous laceration or extension beyond the episiotomy incision was documented.

The sample size was determined using the single population proportion formula, taking a prevalence of 40% from a previous study conducted at St. Paul Hospital, a 95% confidence level, and a margin of error of 5%. Because the source population was less than 10,000, finite population correction was applied, yielding a final sample size of 306. Eligible medical records were selected using systematic sampling. The sampling interval was obtained by dividing the total number of eligible deliveries during the study period by the required sample size. After selecting the first chart randomly from the first sampling interval, every 14th eligible medical record was reviewed until the final sample size was reached. Records with incomplete or missing key clinical information were excluded from the analysis.

Data were collected using a structured chart review checklist adapted from previous literature. The checklist was used to extract information on sociodemographic characteristics, obstetric variables, neonatal characteristics, and maternal outcomes potentially related to episiotomy practice. A pretest was conducted before the actual data collection, and the tool was revised accordingly to improve clarity and completeness. Data collectors were trained on the objectives of the study, the content of the checklist, and the chart review procedure before data collection. The structured chart review checklist used for data extraction is provided in Table A1.

The collected data were checked for completeness, cleaned, coded, and entered into SPSS Version 23.0 for analysis. Descriptive statistics, including frequencies, percentages, means, and ratios, were used to summarize the data. A chi‐square bivariable association analysis was performed to assess the association between episiotomy and each explanatory variable, and crude odds ratios with 95% confidence intervals were calculated. Because several of the candidate predictors are clinically interrelated, all variables with a bivariable *p* value of less than 0.25 were entered together into a multivariable binary logistic regression model, using the enter method, in order to identify factors independently associated with episiotomy. Adjusted odds ratios with 95% confidence intervals were reported for the final model. Multicollinearity among the independent variables was assessed using variance inflation factors, model fit was examined using the Hosmer–Lemeshow goodness‐of‐fit test, and the explanatory power of the model was summarized using the Nagelkerke *R*‐squared statistic. Differences in perineal outcomes between women who did and did not undergo episiotomy were compared using the chi‐square test with corresponding odds ratios. Statistical significance was declared at a *p* value of less than 0.05.

Ethical approval was obtained from the Ethical Review Committee of the School of Medicine. Permission to conduct the study was secured from Wolaita Sodo University Comprehensive Specialized Hospital through the appropriate administrative channels. As the study involved retrospective review of medical records, all extracted information was kept strictly confidential and used solely for research purposes. Personal identifiers were not recorded.

## 3. Results

### 3.1. Prevalence of Episiotomy

A total of 306 records of women who gave birth vaginally were included in the analysis. Among the 306 women included in the study, 141 underwent episiotomy, yielding an overall prevalence of 46.1%. The remaining 165 women (53.92%) delivered without episiotomy. This indicates that episiotomy was performed in nearly one out of every two vaginal births at the study hospital during the study period.

### 3.2. Sociodemographic and Obstetric Characteristics of Study Participants

Most participants were aged 20–34 years, accounting for 239 women (78.10%), whereas 44 (14.38%) were younger than 20 years and 23 (7.52%) were older than 34 years. A total of 192 women (62.75%) had at least one antenatal care follow‐up, whereas 114 (37.25%) had no documented follow‐up. Regarding gestational age at delivery, 252 women (82.35%) delivered at term, 35 (11.44%) delivered before 37 completed weeks, and 19 (6.21%) delivered after 42 weeks. Spontaneous labor was observed in 243 women (79.41%), followed by induced labor in 36 (11.76%) and augmented labor in 27 (8.82%). Primiparous women represented 181 cases (59.15%), whereas 125 (40.85%) were multiparous. Other diagnosed comorbidity was present in 46 women (15.03%). The duration of the second stage of labor was less than 1 h in 146 women (47.71%), 1–2 h in 155 women (50.65%), and more than 2 h in five women (1.63%). Most newborns, 257 (83.99%), had a birth weight of 2500–4000 g, whereas 38 (12.42%) weighed 1500–2499 g and 11 (3.59%) weighed more than 4000 g. The mean Apgar score was 7.0 ± 1.1 at 1 min and 8.7 ± 0.8 at 5 min. These sociodemographic and obstetric characteristics are summarized in Table 1.

**Table 1:** Sociodemographic and obstetric characteristics of women who gave birth vaginally at Wolaita Sodo University Comprehensive Specialized Hospital, Ethiopia, from January 1 to February 28, 2023 (*n* = 306).

Variable	Category	*n*	%
Age	< 20 years	44	14.38
	20–34 years	239	78.1
	> 34 years	23	7.52

Had at least one ANC follow‐up	Yes	192	62.75
	No	114	37.25

Gestational age at delivery	< 37 weeks	35	11.44
	37–42 weeks	252	82.35
	> 42 weeks	19	6.21

Onset of labor	Spontaneous	243	79.41
	Induced	36	11.76
	Augmented	27	8.82

Parity	Primiparous	181	59.15
	Multiparous	125	40.85

Other diagnosed comorbidity	Yes	46	15.03
	No	260	84.97

Duration of second stage of labor	< 1 h	146	47.71
	1–2 h	155	50.65
	> 2 h	5	1.63

Birth weight	1500–2499 g	38	12.42
	2500–4000 g	257	83.99
	> 4000 g	11	3.59

### 3.3. Maternal and Intrapartum Outcomes

Perineal tear was documented in 153 women (50.00%), whereas the remaining 153 women (50.00%) had no recorded tear. Across all vaginal births, first‐degree tears accounted for 102 cases (33.33%) and second‐degree tears for 51 cases (16.67%). No third‐ or fourth‐degree tear was recorded. Meconium‐stained amniotic fluid was documented in 45 deliveries (14.71%). When perineal outcomes were examined by episiotomy status, 77 of the 141 women who underwent episiotomy (54.61%) had a documented perineal tear, compared with 76 of the 165 women who did not (46.06%); this difference was not statistically significant (unadjusted OR = 1.41, 95% CI: 0.90–2.21, *p* = 0.169). Episiotomy was not associated with a lower unadjusted prevalence of documented perineal tear. Second‐degree tears were more frequently documented among women who underwent episiotomy; however, this comparison was unadjusted and may reflect confounding by indication (32 of 141, 22.70%, versus 19 of 165, 11.52%; unadjusted OR = 2.26, 95% CI: 1.21–4.19, *p* = 0.014). First‐degree tear was similarly distributed (31.91% versus 34.55%). A maternal complication other than perineal tear was documented in 21 women (6.86%), comprising maternal exhaustion in eight, wound complication in three, postpartum hemorrhage in three, and other complications in seven; the frequency of these complications did not differ significantly between women who did and did not undergo episiotomy (5.67% versus 7.88%, *p* = 0.594). Overall maternal and selected intrapartum outcomes are presented in Table 2, and the distribution of perineal tears is shown in Figure 1.

**Table 2:** Maternal and selected intrapartum outcomes among women who gave birth vaginally at Wolaita Sodo University Comprehensive Specialized Hospital, Ethiopia, from January 1 to February 28, 2023 (*n* = 306).

Variable	Category	*n*	%
Perineal tear after vaginal birth	Yes	153	50.0
	No	153	50.0
Degree of tear among all vaginal births	First degree	102	33.33
	Second degree	51	16.67
Meconium‐stained amniotic fluid	Yes	45	14.71
	No	261	85.29

**Figure 1:**
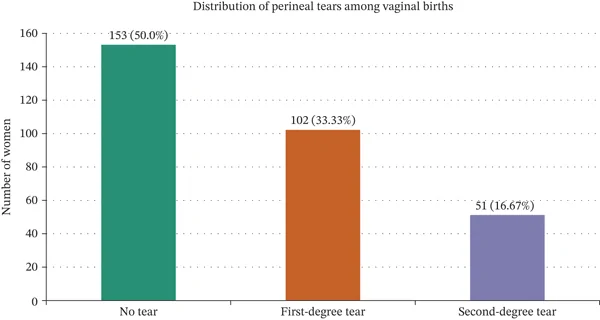
Distribution of perineal tears among women who gave birth vaginally at Wolaita Sodo University Comprehensive Specialized Hospital, Ethiopia, from January 1 to February 28, 2023.

### 3.4. Indications for Episiotomy

An indication was documented for all 141 episiotomies performed during the study period. The most frequent was prolonged second stage of labor, recorded in 51 procedures (36.17%), followed by rigid perineum in 32 (22.70%), fetal compromise in 29 (20.57%), suspected large fetal size in 18 (12.77%), and the need to expedite delivery in 11 (7.80%).

### 3.5. Factors Associated With Episiotomy

In bivariable analysis, parity, antenatal care follow‐up, duration of the second stage of labor, gestational age at delivery, birth weight, and meconium‐stained amniotic fluid were significantly associated with episiotomy. Episiotomy was more common among primiparous women than among multiparous women (62.98% versus 21.60%, *p* < 0.001). Women without antenatal care follow‐up also had a higher proportion of episiotomy than those with at least one follow‐up visit (55.26% versus 40.63%, *p* = 0.018). Similarly, episiotomy was more frequent among women whose second stage of labor lasted at least 1 h compared with those whose second stage lasted less than 1 h (59.38% versus 31.51%, *p* < 0.001). A significant difference was observed across gestational age groups, with the lowest proportion among preterm births (8.57%) and the highest among postterm births (89.47%) (*p* < 0.001). Episiotomy was less frequent among newborns weighing 1500–2499 g than among those weighing 2500 g or more (18.42% versus 50.00%, *p* < 0.001). In addition, episiotomy was more common in deliveries complicated by meconium‐stained amniotic fluid than in those without meconium staining (73.33% versus 41.38%, *p* < 0.001). Other diagnosed comorbidity showed a borderline association, episiotomy being less frequent among women with a documented comorbidity than among those without (32.61% versus 48.46%, COR = 0.51, 95% CI: 0.27–1.00, *p* = 0.068). Maternal age, onset of labor, and time of delivery were not significantly associated with episiotomy. The complete bivariable findings are presented in Table 3, with selected proportions illustrated in Figure 2.

**Table 3:** Bivariable analysis of factors associated with episiotomy among women who gave birth vaginally at Wolaita Sodo University Comprehensive Specialized Hospital, Ethiopia, from January 1 to February 28, 2023 (*n* = 306).

Variable	Category	Episiotomy		Episiotomy %	Crude OR (95% CI)	P value
		Yes, *n*	No, *n*			
Maternal age	20–34 years	112	127	46.86	Reference	0.525
	< 20 years	21	23	47.73	1.04 (0.54–1.97)	0.525
	> 34 years	8	15	34.78	0.60 (0.25–1.48)	0.525
Parity	Primiparous	114	67	62.98	Reference	< 0.001
	Multiparous	27	98	21.60	0.16 (0.10–0.27)	< 0.001
Had at least one ANC follow‐up	Yes	78	114	40.62	Reference	0.018
	No	63	51	55.26	1.81 (1.13–2.88)	0.018
Onset of labor	Spontaneous	117	126	48.15	Reference	0.138
	Induced	11	25	30.56	0.47 (0.22–1.01)	0.138
	Augmented	13	14	48.15	1.00 (0.45–2.22)	0.138
Other diagnosed comorbidity	No	126	134	48.46	Reference	0.068
	Yes	15	31	32.61	0.51 (0.27–1.00)	0.068
Duration of second stage of labor	< 1 h	46	100	31.51	Reference	< 0.001
	≥ 1 h	95	65	59.38	3.18 (1.98–5.09)	< 0.001
Gestational age at delivery	37–42 weeks	121	131	48.02	Reference	< 0.001
	< 37 weeks	3	32	8.57	0.10 (0.03–0.34)	< 0.001
	> 42 weeks	17	2	89.47	9.20 (2.08–40.66)	< 0.001
Birth weight	1500–2499 g	7	31	18.42	Reference	< 0.001
	≥ 2500 g	134	134	50.00	4.43 (1.88–10.41)	< 0.001
Presence of meconium	Yes	33	12	73.33	Reference	< 0.001
	No	108	153	41.38	0.26 (0.13–0.52)	< 0.001
Time of delivery	Daytime	49	68	41.88	Reference	0.511
	Evening	39	41	48.75	1.32 (0.75–2.34)	0.511
	After midnight	53	56	48.62	1.31 (0.78–2.22)	0.511

*Note:* For each variable, one category was selected as the comparison baseline and labeled. Reference, meaning its odds ratio is 1.00. The crude odds ratios shown for the other categories are interpreted relative to that reference category. Episiotomy percent was calculated within each category as yes divided by total times 100. For variables with more than two categories, the *p* value shown in the first row is the overall bivariable *p* value for that variable, whereas the crude odds ratios for the remaining rows compare each category with the stated reference category.

**Figure 2:**
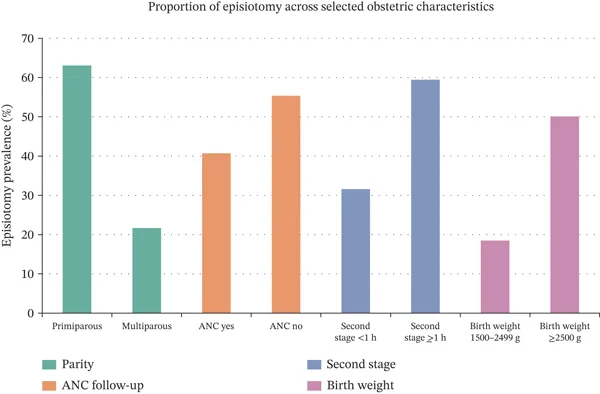
Proportion of episiotomy across selected obstetric characteristics among women who gave birth vaginally at Wolaita Sodo University Comprehensive Specialized Hospital, Ethiopia, from January 1 to February 28, 2023.

### 3.6. Independent Predictors of Episiotomy

All variables with a bivariable *p* value of less than 0.25 were entered together into a multivariable binary logistic regression model. These were parity, antenatal care follow‐up, onset of labor, other diagnosed comorbidity, duration of the second stage of labor, gestational age at delivery, birth weight, and meconium‐stained amniotic fluid. Maternal age (*p* = 0.525) and time of delivery (*p* = 0.511) did not meet the entry criterion and were not included. The model was statistically significant (likelihood ratio chi − square = 130.33, *d* *f* = 10, *p* < 0.001), showed good explanatory power (Nagelkerke *R*‐squared = 0.463), and correctly classified 76.5% of cases. There was no evidence of multicollinearity, with all variance inflation factors at or below 1.08, and the Hosmer–Lemeshow test indicated adequate model fit (chi − square = 4.54, *d* *f* = 8, *p* = 0.806).

Seven of the eight variables entered remained independently associated with episiotomy. Multiparous women had lower odds of episiotomy than primiparous women (AOR = 0.18, 95% CI: 0.10–0.33). Women with no antenatal care follow‐up had higher odds than those with at least one visit (AOR = 1.98, 95% CI: 1.10–3.57), and women with another diagnosed comorbidity had lower odds than those without (AOR = 0.40, 95% CI: 0.17–0.93). A second stage of labor lasting 1 h or longer was associated with almost three times the odds of episiotomy compared with a shorter second stage (AOR = 2.70, 95% CI: 1.52–4.81). Compared with birth at 37–42 weeks, preterm birth was associated with lower odds (AOR = 0.10, 95% CI: 0.03–0.35) and postterm birth with higher odds (AOR = 9.73, 95% CI: 1.92–49.16). A birth weight of 2500 g or more was associated with higher odds than a birth weight of 1500–2499 g (AOR = 3.46, 95% CI: 1.25–9.57) and the absence of meconium‐stained amniotic fluid with lower odds than its presence (AOR = 0.26, 95% CI: 0.11–0.65). Onset of labor was not independently associated with episiotomy (overall *p* = 0.477). Crude and adjusted estimates are presented side by side in Table 4.

**Table 4:** Bivariable and multivariable logistic regression analysis of factors associated with episiotomy among women who gave birth vaginally at Wolaita Sodo University Comprehensive Specialized Hospital, Ethiopia, from January 1 to February 28, 2023 (*n* = 306).

Variable	Category	Crude OR (95% CI)	Adjusted OR (95% CI)	Adjusted P value
Parity	Primiparous	Reference	Reference	—
	Multiparous	0.16 (0.10–0.27)	0.18 (0.10–0.33)	< 0.001
Had at least one ANC follow‐up	Yes	Reference	Reference	—
	No	1.81 (1.13–2.88)	1.98 (1.10–3.57)	0.022
Onset of labor	Spontaneous	Reference	Reference	—
	Induced	0.47 (0.22–1.01)	0.58 (0.22–1.52)	0.266
	Augmented	1.00 (0.45–2.22)	0.74 (0.29–1.89)	0.531
Other diagnosed comorbidity	No	Reference	Reference	—
	Yes	0.51 (0.27–1.00)	0.40 (0.17–0.93)	0.033
Duration of second stage of labor	< 1 h	Reference	Reference	—
	≥ 1 h	3.18 (1.98–5.09)	2.70 (1.52–4.81)	< 0.001
Gestational age at delivery	37–42 weeks	Reference	Reference	—
	< 37 weeks	0.10 (0.03–0.34)	0.10 (0.03–0.35)	< 0.001
	> 42 weeks	9.20 (2.08–40.66)	9.73 (1.92–49.16)	0.006
Birth weight	1500–2499 g	Reference	Reference	—
	≥ 2500 g	4.43 (1.88–10.41)	3.46 (1.25–9.57)	0.017
Presence of meconium	Yes	Reference	Reference	—
	No	0.26 (0.13–0.52)	0.26 (0.11–0.65)	0.004

*Note:* Crude odds ratios are reproduced from Table 3. Adjusted odds ratios are derived from a single multivariable binary logistic regression model containing all of the variables listed. Maternal age and time of delivery did not meet the *p* < 0.25 entry criterion and were not included in the model. Statistical significance was declared at a *p* value of less than 0.05.

Abbreviations: ANC, antenatal care; AOR, adjusted odds ratio; CI, confidence interval; OR, odds ratio.

## 4. Discussion

This study found that the prevalence of episiotomy among women who gave birth vaginally at Wolaita Sodo University Comprehensive Specialized Hospital was 46.1% (141/306). This prevalence is high and indicates that episiotomy remains a common obstetric practice in the study setting. The finding suggests that the procedure may still be used more frequently than would be expected under a restrictive episiotomy approach, which recommends selective rather than routine use [4, 7]. In practical terms, this pattern may reflect institutional practice norms, provider preference, concern about perineal trauma, or attempts to expedite difficult labor, particularly in women perceived to be at increased obstetric risk [15, 16, 19].

The present finding is broadly comparable with reports from other Ethiopian hospital‐based studies showing that episiotomy remains commonly practiced, although the magnitude varies across settings [19–21, 23, 24]. This similarity may be explained by shared clinical environments, comparable provider training backgrounds, and similar labor management practices across hospital settings. At the same time, the prevalence observed in this study remains important because a high episiotomy rate raises questions about whether the procedure is consistently being applied for clear clinical indications or whether it is still used routinely in some circumstances [4, 7, 16, 23].

Whens assessing independent predictors in the multivariable model, parity was one of the strongest factors associated with episiotomy. Multiparous women had markedly lower odds of episiotomy compared with primiparous women (AOR = 0.18, 95% CI: 0.10–0.33). This is clinically plausible, because first‐time mothers are more likely to have a less distensible perineum, a longer labor, and greater provider concern about spontaneous perineal trauma. Other maternal factors were also influential: Women with no documented antenatal care follow‐up had higher odds (AOR = 1.98, 95% CI: 1.10–3.57), potentially reflecting delayed care‐seeking or unrecognized complications.

Intrapartum and fetal characteristics significantly drove intervention. A second‐stage duration of at least 1 h increased the odds of episiotomy almost threefold (AOR = 2.70, 95% CI: 1.52–4.81), likely indicating provider concern over maternal exhaustion or delayed descent. Gestational age at delivery also demonstrated a strong gradient; preterm births had lower odds (AOR = 0.10, 95% CI: 0.03–0.35), whereas postterm births had higher odds (AOR = 9.73, 95% CI: 1.92–49.16). Similarly, birth weight of at least 2500 g was associated with greater odds of episiotomy compared with low birth weight (AOR = 3.46, 95% CI: 1.25–9.57). This may partly reflect the lower likelihood of perineal intervention in births involving smaller infants; the combined category did not permit separate assessment of normal birth weight and macrosomia. Finally, the absence of meconium‐stained amniotic fluid was associated with lower odds of episiotomy (AOR = 0.26, 95% CI: 0.11–0.65), reflecting the urgency that often accompanies meconium‐stained labor.

One association became clear only after adjustment. Women with another diagnosed comorbidity, most often anemia, pregnancy‐induced hypertension, HIV, or gestational diabetes, were less likely to undergo episiotomy (AOR = 0.40, 95% CI: 0.17–0.93). A plausible clinical explanation is that a documented comorbidity prompts closer supervision of labor and a more cautious approach to perineal incision. The subgroup was small, however, so this finding should be regarded as generating a hypothesis rather than settling one.

The pattern of perineal trauma observed in this study deserves particular attention. Perineal tear was documented in 153 of the 306 women (50.0%), comprising 102 first‐degree tears and 51 second‐degree tears. Episiotomy was not associated with a lower unadjusted prevalence of documented perineal tear. Second‐degree tears were more frequently documented among women who underwent episiotomy; however, this comparison was unadjusted and may reflect confounding by indication. Because episiotomy is performed more often in labors already judged to be difficult, part of this difference is likely to reflect confounding rather than harm caused by the incision itself. The finding nevertheless argues against the view that a planned incision reliably prevents uncontrolled perineal injury, supporting evidence that it should be reserved for selected clinical indications [2, 4, 6].

The documented indications add a further layer to this picture. An indication was recorded for every episiotomy performed, which is a strength of record keeping at this hospital. Fetal compromise and the need to expedite delivery accounted for 28.37% of documented indications. However, because indications were recorded as broad categories without sufficient clinical detail, their appropriateness could not be determined retrospectively. Categories such as rigid perineum and suspected large fetal size should be prioritized for standardization and audit. This points to a specific target for quality improvement rather than a general appeal to operate less often.

The care given to the perineal wound after the procedure deserves equal attention. A recent systematic review and meta‐analysis found that lactic acid‐treated wounds healed faster, became infected less often, and were associated with lower pain scores than wounds managed with conventional antiseptics (RR = 0.68, 95% CI: 0.52–0.85) [11]. A subsequent randomized clinical trial supported these findings, demonstrating greater reductions in scar severity and pain scores at 7 and 40 days postpartum for women receiving intravaginal lactic acid gel [12]. These findings are promising but preliminary. Larger multicenter trials and local feasibility and safety assessments are needed before this intervention can be recommended for routine postepisiotomy care in Ethiopia.

These findings point to a need for structured quality improvement. First, a written restrictive episiotomy protocol should be adopted. Second, the hospital should standardize indication lists and audit recorded indications against the protocol. Third, regular institutional audit should be established, with episiotomy rates reported at agreed intervals and broken down by parity and individual provider. Fourth, simulation‐based obstetric training in perineal support and management of the second stage without incision should be offered, supported by continuous professional education [4, 7].

## 5. Limitations of the Study

Although this study provides important evidence, several limitations should be considered. First, the short 2‐month study period (January to February 2023) and the retrospective chart review design meant the analysis depended entirely on existing medical records. Consequently, some variables may have been incompletely documented; for instance, there may have been misclassification of “no ANC” if it merely reflected undocumented antenatal care and possible underdocumentation of third‐ and fourth‐degree tears. Second, the study was conducted in a single comprehensive specialized hospital, limiting generalizability to lower level health facilities where provider experience and case mix may differ. Third, instrumental and complicated deliveries were excluded to maintain a homogeneous population, which may have underestimated variation in episiotomy practice across different delivery circumstances. Finally, operator‐related characteristics and long‐term postpartum outcomes (such as wound healing, pain scores, or pelvic floor function) were not recorded and could not be evaluated, meaning residual confounding cannot be entirely excluded.

## 6. Conclusion

This study found that episiotomy was commonly practiced among women who gave birth vaginally at Wolaita Sodo University Comprehensive Specialized Hospital, with an overall prevalence of 46.1%. The finding indicates that episiotomy remains frequent in the study setting despite recommendations favoring restrictive and selective use. After adjustment in a multivariable model, episiotomy remained independently associated with primiparity, lack of antenatal care follow‐up, absence of another diagnosed comorbidity, a prolonged second stage of labor, gestational age at delivery, birth weight, and meconium‐stained amniotic fluid, suggesting that its use was strongly influenced by identifiable maternal and intrapartum characteristics. The procedure did not prevent perineal trauma, and second‐degree tear was more frequent among the women who received it.

The findings further suggest that episiotomy was more likely to be performed in women perceived to be at increased obstetric risk, particularly primiparous mothers and those with prolonged second stage of labor. However, the relatively high prevalence observed in this study underscores the need to strengthen evidence‐based intrapartum decision‐making and ensure that episiotomy is reserved for clearly justified clinical indications. Overall, the study highlights the importance of promoting selective episiotomy practice as part of improving the quality and safety of maternal care during vaginal delivery.

## 7. Recommendations

Based on the findings of this study, the hospital should strengthen adherence to evidence‐based and restrictive episiotomy practice. Greater institutional emphasis should be placed on ensuring that episiotomy is performed only for appropriate clinical indications rather than as a routine component of vaginal birth care. This may help reduce unnecessary procedures and improve the quality and safety of maternal care.

Healthcare providers should be supported through continued professional training on current intrapartum care standards, including appropriate indications for episiotomy, careful assessment of obstetric risk, and alternative approaches to perineal protection during labor and delivery. Strengthening provider knowledge and clinical decision‐making may help promote more selective use of episiotomy, particularly among primiparous women and in cases involving prolonged second stage of labor.

The hospital should also strengthen clinical audit of episiotomy practice. The indication for each episiotomy is already documented in the delivery record, which provides a sound basis for review; the next step is to compare recorded indications against an agreed protocol and to report episiotomy rates by parity and by individual provider at regular intervals. Documentation should also be extended to postpartum outcomes that were not consistently captured during the study period, including perineal wound infection, wound dehiscence, pain, and length of hospital stay, so that the consequences of the procedure can be monitored alongside its frequency. Routine review of delivery records may further enhance accountability and adherence to recommended standards.

In addition, further research using larger and preferably multicenter study designs is recommended to better understand episiotomy practice and associated maternal outcomes in similar settings. Such studies would be valuable for confirming these findings, improving generalizability, and informing broader clinical and policy recommendations.

## Author Contributions

Conceptualization, N.Y.S. and B.E.D.; methodology, N.Y.S. and D.D.K.; software, N.Y.S. and K.A.B.; validation, N.Y.S., D.D.K., and T.E.L.; formal analysis, N.Y.S. and B.B.B.; investigation, N.Y.S. and B.E.D.; resources, N.Y.S. and B.B.B.; data curation, N.Y.S. and B.E.D.; Writing—original draft preparation, N.Y.S. and B.E.D.; writing—review and editing, N.Y.S., K.A.B., D.D.K., B.B.B., and T.E.L.; visualization, N.Y.S.; supervision, N.Y.S. and B.B.B.; project administration, N.Y.S.

## Funding

No funding was received for this manuscript.

## Ethics Statement

This study was conducted in accordance with the ethical principles of the Declaration of Helsinki. The study used anonymized secondary data obtained from medical records through a retrospective chart review. Because no direct participant contact occurred and no personal identifiers were collected or retained, individual informed consent was not required. Administrative permission to access and review the records was obtained from Wolaita Sodo University Comprehensive Specialized Hospital. Confidentiality was maintained throughout the study by using deidentified data solely for the purpose of this research.

## Conflicts of Interest

The authors declare no conflicts of interest.

## Data Availability

The data that support the findings of this study are available on request from the corresponding author. The data are not publicly available due to privacy or ethical restrictions.

## Appendix A

**Table A1:** Data collection table for episiotomy assessment.

Serial number	1	2	3	4	5	6	7	8	9	10
Patient ID										

Age										

Number of ANC follow‐up										

Gestational age during delivery										

Mechanism of onset of labor										
Spontaneous										
Induced										
Augmented										

Parity										

Other diagnosed comorbidity										
Yes										
No										

Duration of second stage of labor										

Birth weight (g)										
< 1500										
1500–2500										
≥ 2500										

Apgar score										
first min										
fifth min										

Episiotomy										
Yes										
No										

Indication for episiotomy (if performed)										

Perineal tear										
Yes (the degree)										
No										

Delivery outcome(neonate)										

Delivery outcome (mother)										

Other complication										
Yes										
No										

Previous mode of delivery (if applicable)										

Time of delivery										
